# Digital health in pain assessment, diagnosis, and management: Overview and perspectives

**DOI:** 10.3389/fpain.2023.1097379

**Published:** 2023-04-17

**Authors:** Yacine Hadjiat, Lars Arendt-Nielsen

**Affiliations:** ^1^ Paris Saclay University, National Institute of Health and Medical Research, U987, Inserm, Paris, France; ^2^ Center for Neuroplasticity and Pain (CNAP), Department of Health Science and Technology, School of Medicine, Aalborg University, Aalborg E, Denmark; ^3^ Department of Gastroenterology and Hepatology, Mech-Sense, Aalborg University Hospital, Aalborg C, Denmark; ^4^ Steno Diabetes Center North Denmark, Clinical Institute, Aalborg University Hospital, Aalborg, Denmark

**Keywords:** pain, digital health, machine learning, artificial intelligence, digital biomarkers, telehealth, health system, research application

## Abstract

Managing pain is essential for social, psychological, physical, and economic reasons. It is also a human right with a growing incidence of untreated and under-treated pain globally. Barriers to diagnosing, assessing, treating, and managing pain are complicated, subjective, and driven by patient, healthcare provider, payer, policy, and regulatory challenges. In addition, conventional treatment methods pose their own challenges including the subjectivity of assessment, lack of therapeutic innovation over the last decade, opioid use disorder and financial access to treatment. Digital health innovations hold much promise in providing complementary solutions to traditional medical interventions and may reduce cost and speed up recovery or adaptation. There is a growing evidence base for the use of digital health in pain assessment, diagnosis, and management. The challenge is not only to develop new technologies and solutions, but to do this within a framework that supports health equity, scalability, socio-cultural consideration, and evidence-based science. The extensive limits to physical personal interaction during the Covid-19 pandemic 2020/21 has proven the possible role of digital health in the field of pain medicine. This paper provides an overview of the use of digital health in pain management and argues for the use of a systemic framework in evaluating the efficacy of digital health solutions.

## Pain is multi-faceted and must be considered holistically

The pervasiveness of pain across various health conditions and geographies is significant, with an estimated one third of the population impacted (1). The individual, societal and economic cost of pain and sub-optimal or non-treatment is high (2). As many as 40% of people do not receive treatment for the pain they experience (3, 4). Chronic pain affects approximately 20% of the population worldwide and costs between $560–635 billion in the United States alone (5). Pain is a serious health condition, directly impacts quality of life and is a leading cause of suffering globally. For this reason it's treatment is considered t a human rights issue (6). Pain may be acute or chronic (lasting longer than 3 months) (7). The recent revision of the International Classification of Diseases (ICD-11) acknowledges chronic primary pain as multifaceted and persistent, while acknowledging its various aetiologies (8).

In 2020, the International Association for the Study of Pain (IASP) redefined pain as “an unpleasant sensory and emotional experience associated with or resembling that associated with actual or potential tissue damage” (*p*.1976). This definition now clearly recognises that pain occurs in a bio-psychosocial context with its effects will similarly be felt across these dimensions. Specifically, the definition includes that: pain is seen as separate from nociception, the experience of pain is learnt, a patient's reports of pain must be respected, and verbal reports of pain are not necessary for diagnosis (9). Pain, therefore, has a specific physiological cause and is related to *negative emotions such as depression and anxiety; and it impacts functional life activities such as sleep, alertness, attention and cognition*.

The cause and intensity of pain can be physiological, cognitive, or emotional, and treatment paradigms reflect this by including pharmacological, physiological, and psychological interventions (10, 7). An inter-disciplinary approach to pain diagnosis, treatment and management is therefore recommended with the intention of affecting significant clinical and psychosocial effects. Digital health has been one of the most significant additions to this inter-disciplinary approach. The scope of digital health is broad, covering software and hardware applied to health requirements. Digital health includes mobile health, digital technologies in health or applied to health situations, digital devices, telehealth, specialized software, artificial intelligence, machine learning (11). And while efficacy of these interventions is starting to be documented, the introduction of digital health interventions into a framework that does not consider a biopsychosocial approach is a concern (12).

Access to the diagnosis, treatment and management of pain is highly unequal globally, and even within countries linked to socio-economic status. Almost half the world's population is not able to access essential health services (5). The necessity to explore alternative or complementary treatment options is critical to address the burden of pain. To treat pain, one must first start with the diagnosis and measurement of pain. Whilst this may sound uncomplicated, the measurement of pain presents numerous challenges.

## Effective pain assessment and diagnosis is a challenge; innovations must be evidence-based

The assessment of pain includes evaluating the location, frequency, duration, severity, quality, intensity, and unpleasantness. Clearly there is a subjectivity to pain, leading to the primary challenge for its objective measurement. A non-verbal patient, someone who has cognitive deficits, or the very young compound this situation further. It has been necessary therefore to use a variety of measures of pain: (a) self-report using one of the numerous self-report inventories, (b) the observation and infer method, which is useful for non-verbal, the very young, very old, or cognitively and consciously impaired, and (c) indirect physiological or psychophysical indicators. The latter use tools such as electroencephalogram (EEG) and functional magnetic resonance imaging (fMRI) (7) or in some cases quantitative sensory testing; all time consuming, expensive, and not accessible to all. More accessible measurements include their own challenges. Self-report is impacted by psychology and emotion, two essential characteristics of pain which must be equally considered. Levels of consciousness, emotion and inter-rater subjectivity affect observation methods, and the variability of observable pain behaviours differs. Similarly, physiological measures do not have a one-to-one mapping with the intensity of pain and may merely indicate its presence or absence.

Given that measurement should lead to the treatment and management of pain, this challenge of assessment is significant. Rejula et al. (2021) argue that the above challenges demonstrate the necessity for the development or identification of digital biomarkers for pain which would enable quantitative measurement (7). “Digital bio-markers” is a term recently defined to mean the “objective, quantifiable, physiological and behavioral measures collected using digital devices that are portable, wearable, implantable or digestible” (13).

The use of digital health and technology to measure, diagnose and even guide treatment of pain has gained much attention in recent years; and more so during the Covid-19 pandemic (1). The internet and technology are becoming ubiquitous; two thirds of Europe and America having access to the internet. Technology is also becoming more portable. Ubiquity and portability are key determinants to enable digital health solutions to reach scale (14). However, emerging economies do not yet have this level of access and barriers to accessing treatment remain in place. Similarly, diversity in developed economies similarly experience the inequity of digital health solutions (15).

The Covid-19 pandemic played a dual role in the digital health space. On the one hand, it advanced the nature and application of digital health, encouraging healthcare practitioners to use technology to reach patients and adopt new technology and treatment modalities that were not considered mainstream before the pandemic. On the other hand, it also highlighted and entrenched barriers to accessing digital health solutions (16). Based on these experiences, the research areas outlined in this paper are significantly more relevant now and necessary to demonstrate efficacy to ensure the extension of research and development in this area.

There are strong arguments that digital applications for the measurement, diagnosis, management and prevention of pain show promise and impact (3, 8). The extent to which these innovations are affordable, scalable and able to integrate into the healthcare system must be evidenced to avoid the innovation failing or becoming a barrier to further exclusion. It is imperative to evaluate these innovations against a framework for efficacy, scalability, efficiency, and impact. This is especially the case as numerous innovations have been criticised as being unscalable, *ad hoc* pilot projects, and without an evidence base or systematic implementation plan to support them (12).

## Digital health: Promises and potential that require a framework for research and practice

Digital healthcare is an umbrella term used to cover digital medicine and digital therapeutics. Digital health is the broadest term covering lifestyle, wellness and health-related interventions. Digital medicine is a subset of digital health and includes more evidence-based software and hardware. Digital therapeutics is once again a subset of digital health, is also evidence based, with the intention of therapy, remediation, treatment or management of a health condition. While digital health does not require regulatory oversight and approval, digital medicine and digital therapeutics do (17). Digital software and hardware include machine learning, data analytics, big data, applications (apps), and virtual reality. Machine learning (ML) and Artificial Intelligence (AI) can use data, whether self-report or physiological markers, to create algorithms to better understand pain, assess changes to pain, and potentially predict treatment outcomes (8).

Digital therapeutics (DTx) are a type of digital health intervention created using bespoke software and relevant technology to assess, manage and treat medical diseases (18). Best practice, and arguably a gold standard in research, is for digital therapeutics to be evidence-based, and regulator approved (19, 13). (This of course pre-supposes a regulatory or policy model implemented for digital health). Digital therapies may be of two types (or both together): a tracking and monitoring type, and a therapy delivery type. In essence, they include mobile applications, software, digital sensors, and other technology that can monitor certain physiological markers and feed the data back to an algorithm or database to interpret. These methods may not only include feedback mechanisms, but also a form of “push” therapy where treatment is made accessible digitally, for instance, cognitive-behavioural therapy, self-guided relaxation, and immersion tools (7).

**Figure 1:**
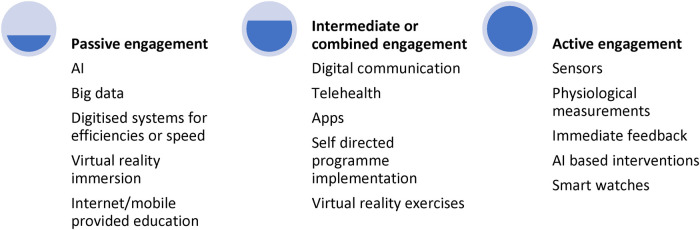
Levels of engagement required from patients in digital health tools.

Digital health therefore falls on a continuum of intervention for its functions of diagnosis, treatment, and management (see Figure 1 below). From the most “passive” level, digital health interventions may include big data analysis within medical settings, extracting trends and treatment options, to chatbots and apps that patients can use when required, all the way up to more “active” and integrated techniques such as physical monitoring, and compulsory engagement of digital tools. There is an argument that the place on the continuum upon which the digital intervention falls, will impact its efficacy and uptake. While passive engagement works well for assessment, active engagement may promote greater interaction and efficacy but only if the patient remains motivated and engaged. Therefore, this continuum must be considered when implementing or scaling a digital health technique or tool (8).

The above levels of engagement are not driven only by the patient but also by the availability, infrastructure, and acceptance of such methods (8), illustrating the need to consider any intervention within a systemic framework.

## Digital health applications must follow best practice principles

Of the many concerns in digital health interventions data privacy is primary—the degree to which personal information is saved and used (3). There are, however, broader concerns. There is also ambiguity about when regulatory approval should be sought, and what basic principles must be followed in the development of big data and algorithms to ensure ethical model development. It is important to ensure that the socio-cultural factors associated with digital interventions in pain are considered, and one should question whether the applications are suitable for integration into insurance and healthcare schemes (12). While the Covid-19 pandemic led to an increase and acceptance of many digital health solutions, these advances should be strengthened by implementing a rigorous framework of evaluation for innovations, to ensure impact, accessibility, acceptance and ethical standards.

Digital therapeutics should not be considered as a replacement for clinical intervention, but rather as a complementary component of intervention, that is evidence-based requiring stringent assessment and approval. The Societal Impact of Pain (SIP) published a white paper calling on the European Union to formalise and acknowledge digital healthcare as an intervention, to support interoperable and validated technologies for pain assessment, to allocate funding to digital health interventions, to ensure that the subjective experience of pain remains part of the assessment of pain, and to establish standards for the sharing of information on evidence-based health technology advances (8).

The proliferation of digital health innovations for the impactful treatment and management of pain is welcomed but should be considered within a systemic framework. Factors such as the efficacy of the application, the scalability or affordability of the application, the regulatory approvals (13) required and the extent to which these applications are complementary and sustainable with other protocols, must be considered to ensure the efforts are beneficial, have integrity, and are able to integrate into healthcare systems. Terhorst et al. (2021) conducted research on mobile apps made available to the public with no framework of approval or vetting in place. The researchers considered the pain apps available in Europe and evaluated their privacy features, usability and functionality, and content quality. The authors found significant data privacy concerns and found little to no scientific evaluation with 99.5% not evaluated using randomised control trials. In this sense the potential power of pain apps is being exploited (3). Day and colleagues (2022) assessed the clinical robustness of the claims of digital health start-ups in the United States. Robustness was measured by analysing regulatory filings and clinical trials. Only 20% of companies demonstrated more than 5 out of a possible 10 score on levels of robustness, the remainder were below (20).

There are, however, numerous research studies on using digital health to measure and treat pain that have been scientifically evaluated, and clearly provide evidence for the applications. It must be noted that results from these research studies are varied in terms of efficacy and impact, and there is still some way to go to develop a solid base of evidence. The use of computers and internet-based programmes, as well as smart phone based interventions, have yielded only small to medium impacts on pain (14). Virtual reality has demonstrated significant pain reduction effects, as have cognitive-behavioural interventions using online modules, especially when therapist interventions are included (21). Digital therapeutics have been successfully used to complement treatment in opioid use disorder (22), including an app that assists patients decrease and manage their opioid use (23). A review of published research on the impact of digital interventions on opioid-based treatment programmes found 9 studies that met the criteria of RCTs. The majority of this research focused on virtual reality apps. Eight of the 9 studies found a significant decrease in pain indicating a clear significant impact of digital health interventions in pain management or opioid use reduction (22).

There is evidently much promise and potential in the use of digital health in the field of pain management.

## Examples of digital health applications in pain management

There are too many examples of how digital health is being applied in the field of pain management to be able to outline in one paper. This paper provides selected examples as an illustration of digital health interventions, but these are by no means exhaustive. These examples merely provide a glimpse into the art and science of the possible and should stimulate further research and innovation. As stressed in the above section, the area requires a greater degree of evidence-based research to validate and replicate findings and thus advance the field. This research and application should be undertaken within a framework to enable better comparison and generalisability of research (see below).

The Australian web-based resource “painHealth”, for example, provides evidence-based research sourced in collaboration with academic institutions and the Department of Health to patients to assist in the self-management of musculoskeletal pain (8). This form of pain intervention outlines rehabilitation and exercise for treatment and recovery and has been shown to be as effective as in-person treatment (24).

“Hinge Health” takes the management of musculoskeletal pain further by integrating a clinical care team with technological innovations. A 12-week digital care programme using a randomized controlled trial on patients with chronic knee pain found a significant difference in pain on two measures of pain (25). Importantly, research on the programme includes a 12-week multi-model mobile digital care programme for over 1,000 patients which found a 69% average pain reduction for participants (four times higher than opioids), decreased anxiety and depression, reduction in surgeries, and sustainability and long-term behaviour change in participants (26). Systemically, these results benefited the workplaces in which it was trialled and reduced insurance claims by half (2). The integration of technology that treats and manages pain as part of workplace wellness programmes is an area of additional research that covers both medical and human management sciences. Additional workplace located research would be beneficial to determine potential cost-savings from innovations on offer.

Digital Internet of Things (IoT) devices can measure various physiological indicators (such as heart rate, skin conductivity, posture, temperature, respiration, EMG, ECG, movement, pain sound and R-R interval). The data is available remotely and can be accessed using mobile or internet connected devices. An example of such a device is “Kipuwex” which monitors physiological indicators remotely, allowing information to be tracked in real time, and analysed across distances (5). Similar devices have demonstrated efficacy in fibromyalgia patients using a smartwatch (18).

Having this information available through a device, and not physically measured by a healthcare professional, provides instant access to data. It also enables the use of algorithms indicating when measures interact, can compare data over time, and can be made available on a dashboard for healthcare professionals to see at a glance (5). The usefulness of these physiological measurements arises as they are applied to an algorithm devised by the health care professional, pertaining to the areas of health intervention or concern (5).

“PainChek” is an app with a pain assessment measure. It uses artificial intelligence, facial recognition, and mobile technology to assess pain remotely. Given the subjectivity of pain assessment and the difficulty experienced when people cannot self-report pain as a result of being non-verbal or cognitively declined, the app constitutes an innovative way to tackle these challenges. The app uses indicators of pain such as facial muscle movements and considers behavioural factors supplied by caregivers. Those who can verbalise can input their pain experiences on a numerical rating assessment. The system then calculates an overall pain score and feeds directly into pain management systems (27). A comparison of the “Abbey Pain Scale” and the “PainChek” app assessment in 22 participants across both at rest conditions and post-movement found a significant high correlation of 0.81 (14). The sample size is small, but the results are significant and indicative of promise in this area.

Biofourmis' “PainFocus” solution integrates machine learning and internet connections, and through measuring specific physiological indicators can analyse pain in the individual over time and predict deterioration (19).

A final example of success can be found in virtual reality (VR). VR is developing quickly as an effective form of digital therapeutic as the technology and content improves. The “SnowWorld” virtual reality video game that assisted burn patients with pain is an often-cited example (22), as is “SpiderWorld” (28). Recently, the Food and Drug Administration has approved an applied VR app called RelieVR for treating back pain (29, 30).

## A framework to consider digital health interventions for pain management

In order for digital health interventions to be widely accepted and used in pain management, there is a need to demonstrate scalability, raise awareness among healthcare professionals, the public, policy makers (and legislators) and insurers (22). While Covid-19 provided some of this opportunity, the evidence-based efficacy of interventions beyond telehealth and app-based information sharing is necessary. The imperative of a framework that is able to consider all stakeholder needs within a system aligns well with a design-thinking approach. Design-thinking allows for the inclusion of contextual factors, usability, and relevance—all features of an innovation that is more likely to be effective. Importantly, design thinking involves identifying the need in context, creating an idea, and testing it to refine, implementing the innovation and checking its efficacy. This is an approach that is iterative and user focused (31).

The literature above considers the needs of users in various settings and when combined a proposed framework can be created. The following features should be included as important matters for policy makers, medical strategists and/or healthcare professionals when considering the use of digital health interventions in their populations for pain management:

– **Regulation and policy**: Is the technology evidence-based and does it have regulatory approval? It is necessary to consider the evidence of efficacy, as many technologies exist that are untested, and with little real world based, significant trials conducted (19).
– **Design:** Health innovations are numerous with only a minority effectively implemented. Arguably, using design-thinking improves the efficacy of interventions as it starts from identifying a real-world need from evidence, considering the system in which the need occurs and testing and refinement *in situ* (32).
– **Research rigour**: Is there an intervention protocol or implementation plan provided? In this case, stand-alone pilot examples of digital interventions are useful, but it is hard to apply them in socio-cultural contexts without a systemic implementation plan that includes buy-in and approval from policy makers, healthcare professionals and the community.
– **Cost-benefit analysis or socio-economic evaluation**: Has consideration been given to the time and effort the digital intervention will take compared to that which healthcare workers currently spend with patients? For instance, a VR intervention requires extensive first consultation training and monitoring to implement (8).
– **Change management plan**: Has the digital intervention been sufficiently explained and considered as a complementary practice to a multi-modal approach?
– **Usage**: Rejula et al (2021) note that for a product to be successful a number of requirements need to be considered (7):
  1. Inter-operability—being able to operate across platforms and hardware, integrate software, and use various technologies such as machine learning, AI, analytics, and allow for data sharing.
  2. Socialisation—the ability to exchange patient data with other members.
  3. Integration—is the innovation able to integrate into the patient's lifestyle and way of living, thereby reducing time with a physician?
  4. Quality of evidence—what evidence of efficacy exists, with RDTs and outcomes being imperative considerations. Has the innovation been tested for usability as well as efficacy? Research and the clinical area need to jointly develop criteria to evaluate apps and interventions from a usability, efficacy, and outcomes perspective, also noting cost and accessibility (33). Gaming—gaming principles offer one way of considering design to ensure engagement and motivated use by the patient (7). Digital interventions, often patient administered and maintained, require motivation and commitment from the patient. Certain programmes include elements of motivation or gamification, or smartphone alerts to increase engagement. More research is needed on these elements (14).

An important consideration to include in the above principles comes from Svendson et al.'s (2020) extensive review of barriers or facilitators of digital health interventions. Motivation and support was an important consideration which considers the degree to which health care workers and patients are motivated and committed to use the digital intervention (34).

The FDA has created a Digital Health Centre of Excellence which has started to set out definitions and guidelines on what constitutes a “device” and a “function”. In addition, the portal provides guidance on what policies and legislation would be applicable to specific digital interventions. It does not, at this time, provide direction on how to design, develop and test devices, nor the regulation of software used in medical products (35).

**Figure 2:**
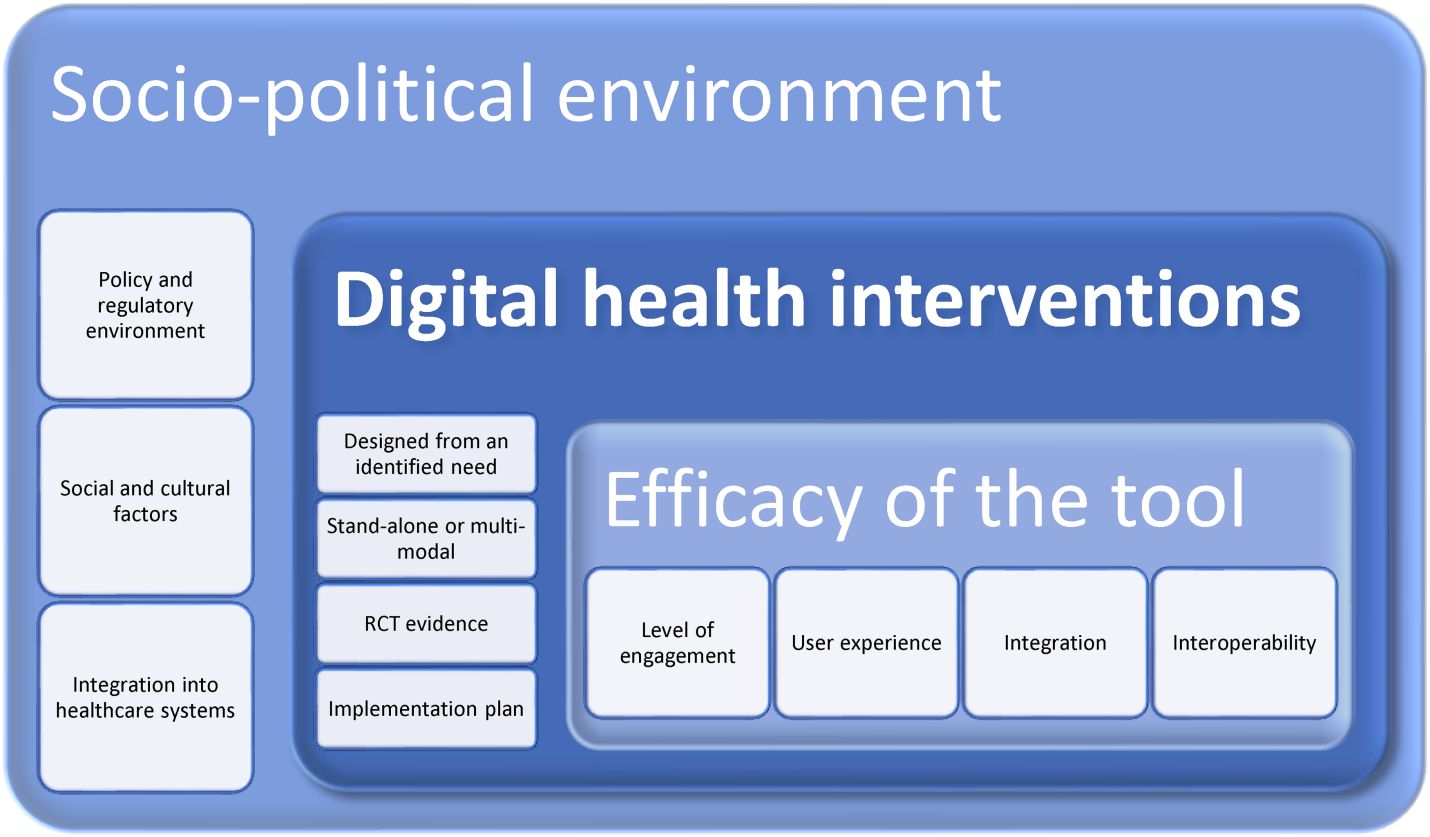
A systemic framework to consider digital health innovations.

Regardless, the above discussions could at this time be displayed as a potential framework for digital health research and practice—see Figure 2 below. This does not presuppose that there are not similar frameworks or models which can be used and adapted or integrated into the below. It is merely a means to ensure the holistic system within which digital health interventions take place are considered. For example, the inclusion of Rejula et al. (2021) characteristics of an effective digital health programme (7). Another example is Ritterband et al.'s behaviour change model for internet interventions that includes metrics of usage and efficacy, change in actions, and treatment support (36). The framework itself will require research and analysis, using a means such as the Delphi Method to garner consensus. As it stands, it is a proposal based on existing literature.

All digital health interventions take place within a broader socio-cultural environment of policy makers and regulators, insurance suppliers and funders. While the innovation itself needs to be assessed for usability, engagement, and sustained use by the individual for it to have impact.

## Conclusion

The assessment, diagnosis, and management of pain is a multi-modal approach (22, 37). For cost efficiency, access and clinical significance, the consideration of including digital health innovations in such an approach is clear. While it is evident that innovations in pain diagnosis, treatment and management using digital technology holds promise, these innovations must be considered against an impact framework, and consideration given to their usability and affordability. The validity and reliability of results should be established through randomised controlled trials and similar evidence-based evaluations should be in place and considered for future developments.

## Author contributions

All authors contributed to the article and approved the submitted version.
